# Sporadic Multifocal Venous Malformations of the Head and Neck

**DOI:** 10.1155/2015/508149

**Published:** 2015-09-21

**Authors:** Michael V. Amato, Neha A. Patel, Shirley Hu, Harry Pantelides

**Affiliations:** ^1^Department of Otolaryngology, New York Medical College, New York, NY 10595, USA; ^2^Department of Otolaryngology, New York Eye and Ear Infirmary of Mount Sinai, New York, NY 10003, USA

## Abstract

*Objective*. To report a case of unusually widespread sporadic venous malformations of the head and neck associated with normal D-dimer levels and, due to the protean clinical manifestations and increased risk of coagulopathy of these lesions, to review their diagnosis and clinical management.* Case Report*. A 25-year-old man presented with a one-year history of intermittent right-sided neck swelling and tongue swelling. Physical exam revealed additional lesions present throughout the head and neck. There was no family history suggestive of heritable vascular malformations. Radiographic imaging demonstrated 15 lesions located in various tissue layers consistent with venous malformations. A coagulation screen showed a normal prothrombin time, activated partial thromboplastin time, international normalized ratio, D-dimer level, and fibrinogen level. It was determined that the patient was not at increased risk for intraoperative coagulopathy and preoperative heparin administration would not be necessary. The patient's buccal and tongue lesions were subsequently excised with no complications. The patient also underwent sclerotherapy evaluation for his neck mass.* Conclusion*. This case describes a unique presentation of sporadic multifocal venous malformations. It also emphasizes the importance of prompt diagnosis and workup when multiple venous malformations are present to prevent morbidity during surgical excision secondary to intravascular coagulopathy.

## 1. Introduction

Vascular malformations are a heterogeneous group of vascular aberrations encompassing a spectrum of subtypes differentiated by vessel type, flow rate, and size [1]. Venous malformations, the most common of these subtypes, are slow-flow, compressible blue masses present at birth which continue to enlarge throughout life. Most cases are unifocal, with extensive venous malformations (MVM) accounting for only one percent of cases and more commonly manifesting as rare, inherited forms [2]. Forty percent of the time, the head and neck regions are involved [3]. The incidence rate for venous malformations is estimated at 1/10,000, with no difference in prevalence between genders [4].

The lesions of MVM tend to be small and typically evade clinical diagnosis until adulthood. However, MVM have been associated with an increased risk of spontaneous thrombosis and thrombolysis, a condition termed localized intravascular coagulopathy (LIC). Severe LIC can potentially progress to disseminated intravascular coagulopathy (DIC) during surgical excision and thus mandates preoperative evaluation [5].

## 2. Case Report

An otherwise healthy 25-year-old male presented with a one-year history of intermittent right-sided neck swelling. He also reported nonspecific right buccal and left ventral tongue masses that had been present for over 8 months. These lesions caused occasional discomfort as well as cosmetic concern. The patient denied any history of similar findings in the past. He denied fevers and pain, ulcers, or bleeding from the sites. Swallowing, speech, and respiratory functions were unaffected. He denied any changes in bowel movements, hematochezia, or melena. There was no family history suggestive of a heritable form of venous malformations.

**Figure 1:**
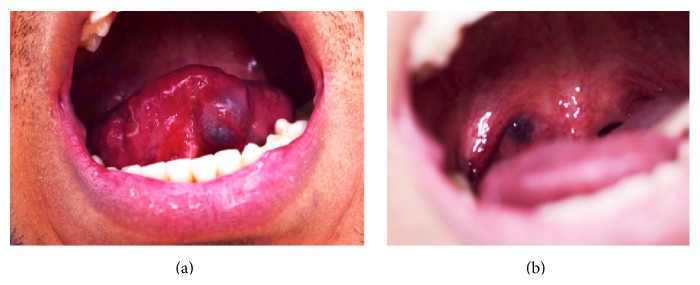
(a) Left ventral tongue lesion. (b) Oropharyngeal lesion located in the right superior tonsillar fossa.

**Figure 2:**
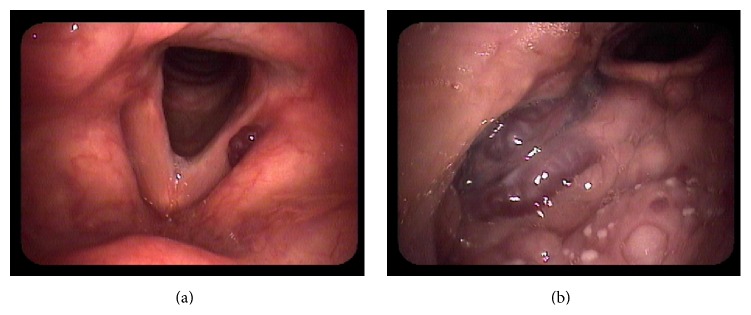
(a) Flexible laryngoscopy showing left laryngeal ventricle lesion. (b) Flexible laryngoscopy showing lesion of the tongue base.

On physical exam, a 3 cm × 3 cm area mass was noted on the right side of the neck at level 2. The area was soft and nontender and had no associated skin changes. A thorough dermatological exam yielded no additional cutaneous lesions. A 2 cm right buccal lesion and a 3 cm ventral tongue lesion were observed on inspection of the oral cavity (Figure 1(a)). Both were soft, nontender, relatively well-circumscribed, easily compressible, and associated with bluish mucosal change. The patient had similar 5 mm lobulated lesions located in the right posterior tongue and right superior tonsillar fossa (Figure 1(b)). Nasal endoscopy further revealed a 5 mm lesion in his left nasopharynx, and flexible laryngoscopy showed a 3 mm lesion in the left laryngeal ventricle (Figure 2(a)) and a 1 cm lesion in the right tongue base (Figure 2(b)).

**Figure 3:**
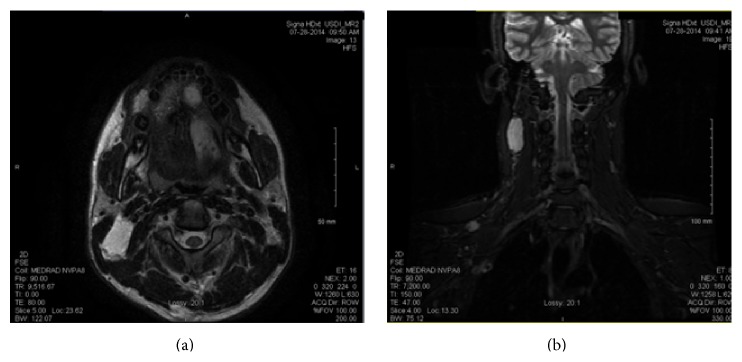
(a) Axial T2-weighted MRI demonstrating soft tissue masses with hyperintense signal of the left tongue and right submandibular triangle. (b) Coronal T2-weighted MRI demonstrating hyperintense lesions of the right suprahyoid posterior triangle and right supraclavicular fossa.

T2-weighted magnetic resonance imaging (MRI) of the neck demonstrated the presence of 15 well-defined, variably sized, hyperintense lesions involving multiple tissues including the skin, mucosa, muscle, and viscera throughout the head and neck which were consistent with venous malformations. Lesions were found in the thyroid gland, left floor of the mouth, right suprahyoid posterior triangle, right posterior submandibular triangle, right supraclavicular fossa, and right lateral chest wall and superficial to the right superior trapezius (Figures 3(a) and 3(b)). Importantly, these lesions had definitive borders either with no drainage or with low draining veins. A Doppler ultrasound of the right side of the neck demonstrated a 3.7 cm × 1.3 cm × 2.7 cm lesion deep to the subcutaneous fat with rare, minute foci of vascular flow. Computed tomography (CT) of the neck did not reveal any phleboliths.

The patient had prior blood work confirming HIV-negative status and a negative tuberculin skin test. A basic metabolic panel and complete blood count were unremarkable. Importantly, hematological investigations demonstrated a normal prothrombin time, activated partial thromboplastin time, international normalized ratio, fibrinogen level (219 mg/dL), and *D*-dimer level (0.53 mcg/mL). The right buccal lesion was subsequently biopsied, confirming a CD31-immunoreactive, M2A-negative, and GLUT1-negative vascular lesion.

Management involved a multidisciplinary team consisting of a head and neck surgeon, a vascular malformation specialist, and a hematologist. Since the patient was not at an increased risk for intraoperative coagulopathy and given the superficial location, relatively small size, and normal venous drainage of the buccal and tongue lesions, complete surgical excision of these lesions was recommended. Heparin was not given preoperatively. The excisions were performed meticulously with no residual lesion, and an intraoperative frozen section confirmed the diagnosis of venous malformation. The patient was referred separately for sclerotherapy evaluation of the neck mass. The decision was made to observe and follow the remaining lesions for any clinical or symptomatic changes. In addition, the patient received counseling on molecular genetic testing, but he was subsequently lost to follow-up.

## 3. Discussion

Vascular malformations are slow-flow collections of ectatic vessels that are present at birth, grow throughout life, and do not involute spontaneously. Most lesions are occult in nature, as seen with our patient, but may manifest in adulthood as progressive swelling secondary to infection, trauma, or the hormonal changes of puberty or pregnancy [6]. Depending on the location and size of the lesions, patients may report cervical swelling, pain, bleeding, dysphonia, dysphagia, stertor, stridor, or airway compression [3]. As the lesions progress, they can develop thrombolites, palpable intralesional calcifications often discovered on X-ray or CT. None were present in this case.

Venous malformations are most commonly sporadic and unifocal, but MVM may occur and are often associated with a number of autosomal dominant genetic vascular anomalies, such as multiple cutaneous and mucosal venous malformations (VMCM) and glomuvenous malformations (GVM) [5]. Disordered vasculogenesis results from mutations in the receptor tyrosine kinase TIE2 (or* TEK*) on chromosome 9p21 in VMCM and loss-of-function mutations in glomulin on chromosome 1p21-22 in GVM [7]. Limaye et al. [8] concluded that sporadic disease might similarly be explained by somatic mutations in* TEK*. A thorough record of family history and possible genetic testing are thus key parts of the clinical evaluation. In this case report, the patient presented with disseminated multifocal lesions, no apparent family history, and normal *D*-dimer and fibrinogen levels. This constellation of findings has not yet been reported in the literature. Unfortunately, molecular genetic studies were not completed on the tissue, and it remains unknown whether a pathogenic variant in* TEK* is the etiology for this patient's MVM.

Accounts of severe coagulopathies with MVM have been described and are frequently associated with high *D*-dimer levels, low fibrinogen levels, and extensive lesions of larger size and deeper tissue involvement [2, 5]. Spontaneous intralesional thrombosis and subsequent activation of the coagulation pathway lead to increased *D*-dimer fragments, a proposed biomarker for both venous malformations and the risk of coagulopathy [9]. This condition, LIC, predisposes the affected individual to developing life-threatening DIC during surgical intervention. LIC occurs in one-half of patients with MVM. The increased risk of DIC necessitates a thorough preoperative assessment for subclinical coagulopathy with a coagulation panel and measurement of *D*-dimer levels. Patients with important or severe LIC, defined as *D*-dimer levels twice the upper limit of normal, require Low Molecular Weight Heparin started 10 days before surgery to prevent catastrophic intraoperative hemorrhage [2, 5].

Venous malformations are diagnosed clinically with a full head and neck examination and laryngoscopy and are subsequently confirmed and evaluated for origin and regional extension with imaging [3]. Doppler ultrasonography can identify characteristic absent-to-low venous blood flow dynamics that differentiate venous malformations from other high flow vascular malformations. MRI is the most sensitive imaging modality for assessment of regional extension, while CT can detect calcified phleboliths. Direct percutaneous phlebography is reserved for the diagnostic confirmation of atypical presentations [3] and vascular visualization during sclerotherapy [5].

Treatment is dependent on the size and location of the malformations and the presenting symptoms. Small, painless lesions may not necessitate intervention, but larger symptomatic malformations require medical or surgical management. Minor and superficial well-circumscribed malformations of the head and neck can be treated with surgical excision, which involves careful removal of the skin and mucosa to prevent reoccurrence, or Nd:YAG laser treatments [10, 11]. Surgery remains an effective and even curative option for localized and superficial or compound venous malformations [12]. More extensive malformations can be successfully reduced with percutaneous sclerotherapy [5], utilizing intralesional injections of ethanol-based gels and even bleomycin with the proper precautions [13]. However, the development of multiple thromboses following sclerotherapy for MVM has been reported [14]. Very severe and extensive malformations are typically treated with a multimodal strategy of surgical management augmented with sclerotherapy [6].

Few reports in the English literature have described sporadic MVM, and none have described a normal *D*-dimer level with the degree of involvement that is seen in this case [6]. Although MVM are rare and often asymptomatic, the risk of occult coagulopathy associated with widespread lesions demands increased clinical awareness, by pediatricians and otolaryngologists alike, for the associated risks, diagnosis, and management of venous malformations. A complete coagulation panel and evaluation of regional extension are necessary before any surgical manipulation to assess for the risk of LIC and catastrophic intraoperative hemorrhage. Surgical excision remains a superior option and may offer a cure when there is a minimal risk of LIC and the lesions are superficial in the skin or mucosa.

## 4. Summary

(1) Venous malformations are slow-flow vascular anomalies with protean clinical manifestations that can present focally or diffusely. We report a rare case of sporadic MVM with normal *D*-dimer levels.
(2) Severe coagulopathies such as LIC and DIC are associated with MVM, especially when the *D*-dimer level is elevated.
(3) The increased risk of severe coagulopathy necessitates a thorough preoperative assessment for subclinical coagulopathy and preventive treatment with heparin as necessary before intervention is determined.

## References

[1] Mulliken J. B., Glowacki J. (1982). Hemangiomas and vascular malformations in infants and children: a classification based on endothelial characteristics. *Plastic and Reconstructive Surgery*.

[2] Dompmartin A., Vikkula M., Boon L. M. (2010). Venous malformation: update on aetiopathogenesis, diagnosis and management. *Phlebology*.

[3] Dubois J., Soulez G., Oliva V. L., Berthiaume M.-J., Lapierre C., Therasse E. (2001). Soft-tissue venous malformations in adult patients: imaging and therapeutic issues. *RadioGraphics*.

[4] Brouillard P., Vikkula M. (2007). Genetic causes of vascular malformations. *Human Molecular Genetics*.

[5] Dompmartin A., Acher A., Thibon P. (2008). Association of localized intravascular coagulopathy with venous malformations. *The JAMA Dermatology*.

[6] Chava V., Shankar A., Vemanna N., Cholleti S. (2013). Multiple venous malformations with phleboliths: Radiological-pathological correlation. *Journal of Clinical Imaging Science*.

[7] Boon L. M., Mulliken J. B., Vikkula M. (1994). Assignment of a locus for dominantly inherited venous malformations to chromosome 9p. *Human Molecular Genetics*.

[8] Limaye N., Wouters V., Uebelhoer M. (2008). Somatic mutations in angiopoietin receptor gene TEK cause solitary and multiple sporadic venous malformations. *Nature Genetics*.

[9] Dompmartin A., Ballieux F., Thibon P. (2009). Elevated D-dimer level in the differential diagnosis of venous malformations. *Archives of Dermatology*.

[10] Werner J. A., Dünne A.-A., Folz B. J. (2001). Current concepts in the classification, diagnosis and treatment of hemangiomas and vascular malformations of the head and neck. *European Archives of Oto-Rhino-Laryngology*.

[11] Richter G. T., Braswell L. (2012). Management of venous malformations. *Facial Plastic Surgery*.

[12] Richter G. T., Friedman A. B. (2012). Hemangiomas and vascular malformations: current theory and management. *International Journal of Pediatrics*.

[13] Eivazi B., Werner J. A. (2013). Management of vascular malformations and hemangiomas of the head and neck-an update. *Current Opinion in Otolaryngology and Head and Neck Surgery*.

[14] Martin L. K., Russell S., Wargon O. (2009). Chronic localized intravascular coagulation complicating multifocal venous malformations. *Australasian Journal of Dermatology*.

